# Integrated Bioinformatics and Experimental Analysis Identified TRIM28 a Potential Prognostic Biomarker and Correlated with Immune Infiltrates in Liver Hepatocellular Carcinoma

**DOI:** 10.1155/2022/6267851

**Published:** 2022-10-04

**Authors:** Jiyu Han, Yanhong Wang, Haichao Zhou, Songtao Ai, Daqian Wan

**Affiliations:** ^1^Department of Orthopedics, Tongji Hospital, School of Medicine, Tongji University, Shanghai 200065, China; ^2^Key Laboratory of Spine and Spinal Cord Injury Repair and Regeneration, Ministry of Education, Shanghai 200065, China; ^3^Department of Radiology, Shanghai Ninth People's Hospital, Shanghai Jiao Tong University School of Medicine, Shanghai 200011, China

## Abstract

**Background:**

Since the 1970s, liver hepatocellular carcinoma (LIHC) has experienced a constant rise in incidence and mortality rates, making the identification of LIHC biomarkers very important. Tripartite Motif-Containing 28 (TRIM28) is a protein-coding gene which encodes the tripartite motif-containing proteins (TRIMs) family and is associated with specific chromatin regions. TRIM28 expression and its prognostic value and impact on the immune system in LIHC patients are being investigated for the first time.

**Methods:**

The TRIM28 expression data from TCGA database was used to analyze TRIM28 expression, clinicopathological information, gene enrichment, and immune infiltration and conduct additional bioinformatics analysis. R language was used for statistical analysis. TIMER, CIBERSORT, and ssGSEA were used to assess immune responses of TRIM28 in LIHC. Next, the results were validated using GEPIA, ROC analysis, and immunohistochemical staining pictures from the THPA. GSE14520, GSE63898, and GSE87630 datasets were analyzed using ROC analysis to further evaluate TRIM28's diagnostic value. To ultimately determine TRIM28 expression, we performed qRT-PCR (quantitative real-time polymerase chain reaction).

**Results:**

High TRIM28 expression level was associated with T classification, pathologic stage, histologic grade, and serum AFP levels. In patients with LIHC, TRIM28 was an independent risk factor for a poor prognosis. The pathways ligand-receptor interaction, which is critical in LIHC patients, were closely associated with TRIM28 expression, and the function of DC could be suppressed by overexpression of TRIM28. As a final step, our results were validated by GEO data and qRT-PCR.

**Conclusions:**

TRIM28 will shed new light on LIHC mechanisms. As an effective diagnostic and intervention tool, this gene will be able to diagnose and treat LIHC at an early stage.

## 1. Introduction

The incidence and mortality of liver hepatocellular carcinoma (LIHC) have increased over the past 40 years, making it important to identify biomarkers for LIHC [1]. LIHC is being monitored due to concerns about the COVID pandemic and associated policy lockdowns [2]. A recent overview on global cancer statistics released in 2020 revealed that 906,000 new diagnosed cases and 830,000 deaths occurred from LIHC, with more than half occurring in China [3]. Hepatocellular carcinoma (HCC) is the most common histological subtype of primary liver cancer (75-85%) [4]. Now, the development of LIHC is associated with a number of risk factors, including hepatitis B and C, excessive drinking, chemical exposure, tobacco use, and aflatoxin [5–7]. At present, there are noninvasive detection and diagnosis methods for LIHC, but they are not sensitive enough for early detection of LIHC [8]. In order to improve LIHC prognosis, it is important to identify more specific biomarkers and possible treatment targets.

Tripartite Motif-Containing 28 (TRIM28) is a protein-coding gene which encodes the tripartite motif-containing proteins (TRIMs) family and is associated with specific chromatin regions. TRIMs family are linked with autoimmune and autoinflammatory diseases which are closely related to malignant tumor [9]. Meanwhile, previous study shows that TRIM28 plays a critical role in T cell activation and T cell tolerance [10]. So, we hypothesize that TRIM28 is linked to immune cell infiltration and significantly promotes tumor progression in LIHC. The hypothesis is in accordance with the results of these past studies [11–14].

In spite of the fact that various types of cancers, comprising colorectal cancer, melanoma, kidney renal clear cell carcinoma, and lung adenocarcinoma are associated with TRIM28-associated immune responses, there is still a lack of understanding of how TRIM28 contributes to immune infiltration and prognosis in LIHC [15–18]. As a response to this challenge, The Cancer Genome Atlas (TCGA, https://cancergenome.nih.gov/) was used to analyze and check the expression level of TRIM28 in LIHC. Under RStudio 1.4, we used R software (version 3.6.3) to assess the relationship between TRIM28 expression and some clinicopathological parameters, as well as possible prognostic value in LIHC. Gene ontology (GO) analyses, Kyoto Encyclopedia of Genes and Genomes (KEGG) analyses, and protein–protein interaction (PPI) networks were performed to clarify the pathogenic impact of TRIM28 and to understand the regulatory mechanisms that govern LIHC invasion and metastasis. Tumor Immunoassay Resource (TIMER) (https://cistrome.shinyapps.io/timer/), CIBERSORT algorithm, and single sample gene-set enrichment analysis (ssGSEA) were performed to further investigate the relationship between TRIM28 and Tumor-Infiltrating Immune Cells (TIICs). Furthermore, Gene Expression Profiling Interactive Analysis (GEPIA), Kaplan–Meier (K-M) survival analysis (http://kmplot.com/analysis/), and the Human Protein Atlas (THPA) were used to compare and assess the interrelationship between high TRIM28 and poor prognosis. Finally, receiver operating characteristic curve (ROC) and experimental analysis were constructed to determine TRIM28's diagnostic value.

This is the first analysis of the relationship of TRIM28 with LIHC. In order to develop and propagate LIHC, a variety of causative mechanisms and risk factors must be considered in its pathogenesis and development. There is a strong association between higher TRIM28 expression and poor prognosis among the diagnostic criteria, outcome events, and influencing factors. Moreover, GO and KEGG analyses revealed that TRIM28 was involved in appendage development, cell cycle control, amino acid, and fatty acid metabolism. We also explored the correlation between TRIM28 and TIICs. This research investigated the function of TRIM28 in LIHC and explored effective molecules to diagnose and treat LIHC.

## 2. Materials and Methods

### 2.1. Data Acquisition and Mining

The applied data, including clinical data, immune system infiltrates, and gene expression data (workflow type: HTSeq-TPM) were obtained from the TCGA database [19]. Samples will also be excluded from the study if data sources are missing, insufficient, or unclear. Analyzing and investigating the data was based on both RNA-sequences and clinical data which were selected for further study. Our research included 424 samples, 374 of which were LIHC tissues and 50 of which were normal healthy liver tissues. As part of the investigation of mechanisms of TRIM28 expression, LIHC patients were grouped into two groups: those with high or low expression levels of TRIM28. In accordance with the publication guidelines offered by TCGA, we conducted our research [20]. We used the Gene Expression Omnibus (GEO) database to collect 3 gene expression profiling datasets (GSE14520, GSE63898, and GSE87630) to determine the expression and diagnostic value of TRIM28 [21–23].

### 2.2. Validation of TRIM28 Expression

TCGA dataset was analyzed to confirm the potential prognostic role of TRIM28 gene in LIHC. In order to compare TRIM28 gene differences between LIHC samples and normal tissues, independent sample *t*-test was used for nonpaired samples and paired *t*-test was used for paired samples. In order to plot the results, boxplots were generated using the ggplot2 R package.

### 2.3. Survival Analysis Based on TRIM28 Expression

To summarize, survival analysis was performed by graphing K-M survival curves with the R packages survival and survminer. The K-M survival curve was used to compare the OS and progression-free interval (PFI) between the high and low TRIM28 groups. Based on the OS and PFI time, we calculated the relationships between TRIM28 expression level and patients' survival outcomes. Additionally, ROC curves were generated using the R language package pROC to assess further the outcomes of K-M survival analysis [24].

### 2.4. GO and KEGG Pathway Enrichment Analyses

We conducted ssGSEA by normalizing RNA-sequences data [25]. With default parameters, gene-set permutations were set to 1,000. TRIM28 was analyzed using ssGSEA for GO pathway enrichment and KEGG pathway enrichment to determine function. Statistics were considered significant when enrichment results with two conditions ((NOM) *P* value <0.05 and false discovery ratio (FDR) *P* value <0.25) was considered.

### 2.5. Construction of the Predicted PPI Network

In the PPI network, protein complexes are formed either by biochemical events or electrostatic forces, and each complex performs a unique biological function. PPI networks act as an organism's skeleton, allowing it to respond to genetic and environmental signals. By understanding these circuits, we may be able to better predict gene function and cellular behaviour. PPI can be predicted using an online biological tool called STRING that includes direct (physical) as well as indirect (functional) associations [26]. The differentially expressed genes (DEGs) were identified with the help of the PPI database STRING version 11.0. DEGs must meet the following criteria: the threshold values of ∣ log2 fold − change (FC)  | >2.0 and adjusted *P* value (adj. *P* value) <0.05. As a cut-off criterion for significant interactions in this network, a medium confidence score (0.400) was required. Using Cytoscape (version 3.8.2), the network was visualized [27].

### 2.6. Immune Infiltrates Analysis

Infiltrates of immune cells across a variety of tumor types can be systematically analyzed using TIMER, a comprehensive and publicly available resource [28]. TIMER was used for investigating the relationship between the expression of TRIM28 and tumors. As part of this LIHC study, the TIMER correlation module was used to analyze the relationship between tumor-infiltrating immune cells and gene expression profiles. The deconvolution statistical method is used to examine the association between infiltrating immune cells and TRIM28 genes in TIMER. As a result of the gene modules, we examined the correlation between TRIM28 and the abundance of immune infiltration in LIHC. A picture of TRIM28 against tumor purity was drawn by TIMER [29]. CIBERSORT (https://cibersort.stanford.edu/), a deconvolution algorithm by evaluating the expression of related genes based on gene expression, served as examination of the relevance between TRIM28 expression and the infiltration of immune cells in LIHC [30]. To build gene expression datasets, we used standard annotation files with a 1,000 permutation default signature matrix. Based on Markov chain Monte Carlo (MCMC) methods, CIBERSORT calculated the *P* value of the deconvolution method. We split 375 tumor samples into two groups to investigate how TRIM28 expression affects the immune microenvironment. Based on the *P* value <0.05, we identified the types of lymphocytes which were affected by TRIM28. The content of immune cells in LIHC TCGA samples were quantified via ssGSEA package and “GSVA” R package.

### 2.7. Comprehensive Analysis

GEPIA analyzed the expression of RNA-sequencing data of 8587 normal and 9736 tumor samples from public databases (TCGA and GTEx) [31]. Overall survival was analyzed by GEPIA for TRIM28 expression in LIHC. Additionally, to calculate differential expression of TRIM28, boxplots were generated via tumor or normal state. The interaction between TRIM28 expression and LIHC survival information were examined by K-M analysis of survival curves [32]. Using the log-rank *P* value and the hazard ratio (HR), the risk of death was calculated. *P* value <0.05 was counted as statistically significant.

### 2.8. Immunohistochemistry-Based Validation of Hub Genes in THPA

THPA, a database funded by a Swedish grant, was used for finding information of immunohistochemically stained tissues and cells for 26,000 human proteins. Antibody proteomics allows THPA detection of normal and LIHC tissues, which commonly regarded as hub gene validation. In this way, THPA can verify TRIM28 gene in normal tissues and LIHC tissues.

### 2.9. Cell Culture

The human normal liver cell lines (MIHA) and hepatocellular carcinoma cell lines (Hep3B, SMMC7721, and MHCC97H) were purchased from the cell bank of the Institutes for Biological Sciences (Shanghai, China). An STR profiling test was conducted in order to verify the authenticity of cell lines. Cells were cultured in Dulbecco's modified Eagle's medium (DMEM) supplemented with 10% fetal bovine serum (FBS) at 37°C and 5% CO_2_ in a humidified incubator.

### 2.10. Quantitative Reverse-Transcription Polymerase Chain Reaction

According to the manufacturer's instructions, the maximum amount of RNA from the cell lines was extracted using TRIzol reagent (Invitrogen, Thermo Fisher Scientific, Inc.) and reverse transcription was accomplished with the PrimeScript™ RT Reagent kit (Takara, Shiga, Japan). The TRIM28 mRNA expression level was measured by Applied Biosystems® 7500 Fast Real-Time PCR System (Thermo Fisher Scientific, Waltham, MA) and accompanying Applied Biosystems® 7500 Software (version 2.0.6). A gene called GAPDH served as a housekeeping gene. In order to amplify TRIM28, we used forward primer TTTCATGCGTGATAGTGGCAG and reverse primer GCCTCTACACAGGTCTCACAC. The GAPDH sequences of the primers used were forward, GCCTTTGATGACTCAGCTCC and reverse, TTCCTGAAAAGTCACCACCC.

### 2.11. Statistical Analysis

Statistical software R was used for conducting all statistical analyses. Univariate Cox analysis was used to determine the multivariate HR and 95% confidence intervals (95% CI). The R packages rms and survival were used to formulate the nomogram model and calibration curves. After that, we examined how TRIM28 expression and other clinical and pathological features affected OS. The significance threshold was set as probability *P* value <0.05. Logistic regression was used to evaluate the associations between TRIM28 expression and clinical characteristics (T stage, pathologic stage, histologic grade, AFP, OS event, weight, and BMI).

## 3. Results

### 3.1. Survival Outcomes and Variable Analyses

**Figure 1:**
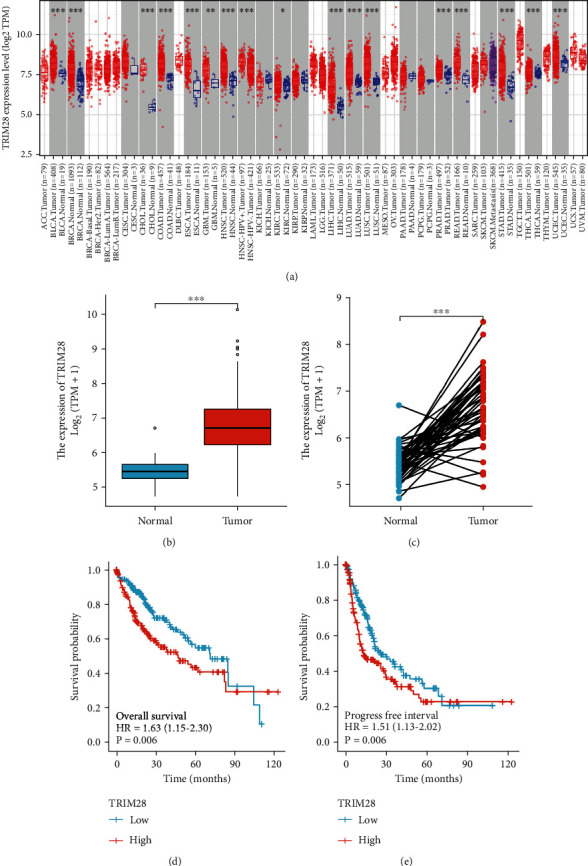
TRIM28 expression in tumors. High TRIM28 expression in LIHC predicts a poor prognosis and serves as an oncogene in tumor disorders. (a) Human TRIM28 expression levels in pancancer from TIMER database. (b) TRIM28 in LIHC samples from TCGA. (c) TRIM28 in paired LIHC samples from TCGA. (d, e) TCGA survival status and TRIM28 expression (^∗^*P* < 0.05, ^∗∗^*P* < 0.01, ^∗∗∗^*P* < 0.001).

Firstly, data was analyzed to confirm TRIM28 expression levels in pancancer via TIMER datasets. According to the analysis, expression levels of TRIM28 are upregulated in most tumors (Figure 1(a)). It has not been studied whether TRIM28 plays a role in human liver cancer, particularly in LIHC. To validate TRIM28's prognostic influence in LIHC, we analyzed the TCGA datasets to determine how TRIM28 expression differed between normal and tumor tissue and discovered that the level of TRIM28 was increased in all LIHC tissues compared to normal liver tissue (Figure 1(b)).

**Figure 2:**
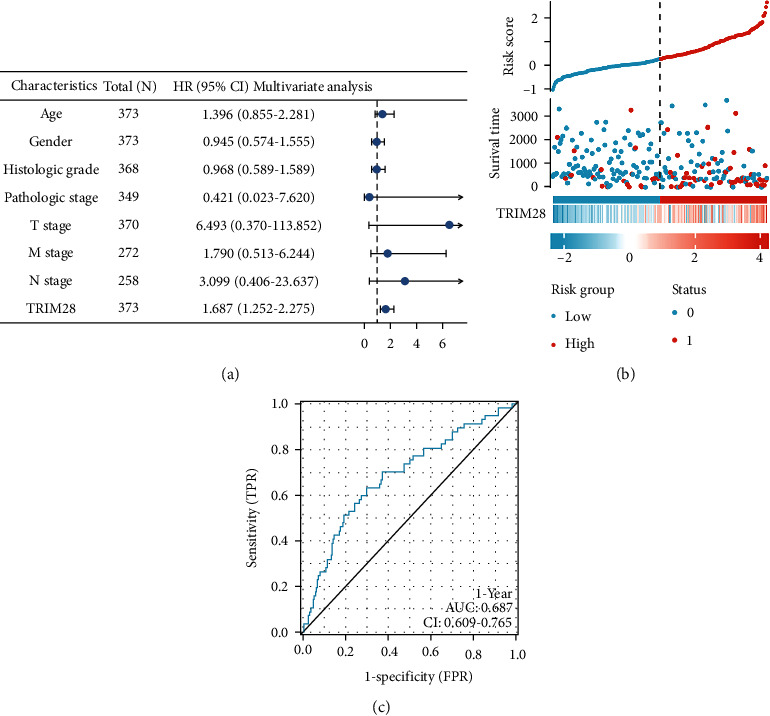
Prognostic analysis of the TRIM28. (a) Multivariate Cox analysis of TRIM28 expression and other clinicopathological variables. (b) TRIM28 expression distribution and survival status. (c) ROC curves of TRIM28.

The same outcome was obtained in paired LIHC tissues compared with normal tissues (*n* = 50) (Figure 1(c)). Additionally, LIHC patients with low TRIM28 expression had better OS and PFI (Figures 1(d) and 1(e)). A Cox analysis was performed as valuing the relationship between TRIM28 expression and OS, as well as other multivariable characteristics in LIHC patients, as shown in Table 1. Based on univariate regression analysis, it appears that pathological stage, T stage, M stage, and OS are highly correlated with TRIM28. Based on the multivariate analysis shown in Figure 2(a), TRIM28 expression (*P* value <0.001) has been found to be an independent predictor of poor prognosis for oncologist-patients (Table 1).  Figure 2(b) shows the expression distribution of TRIM28 as well as survival status and TRIM28 expression profiles for patients with LIHC. In Figure 2(c), the ROC curve found that TRIM28 was associated with prognosis since its AUC for survival prediction was 0.687.

### 3.2. Construction and Prediction of Nomogram Model

**Figure 3:**
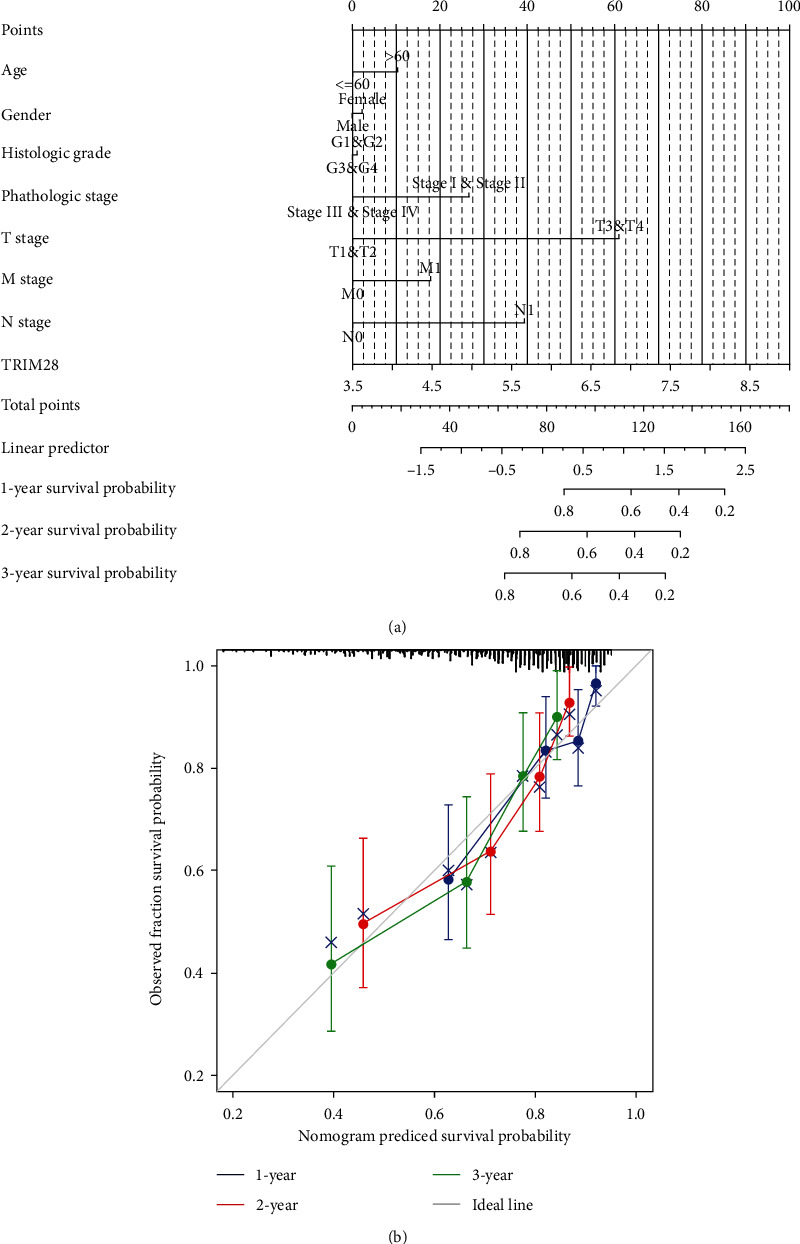
Construction and validation of a prediction model for robust nomogram. (a) Based on the clinicopathological parameters, a nomogram prediction model was built. (b) The calibration curves showed that the nomogram model demonstrated excellent performance for predicting the 1-, 2-, and 3-year OS. OS: overall survival.

In order to establish a clinically applicable way that could assess the prognosis of LIHC patients, the nomogram prediction for predicting the survival probability at 1-, 2-, and 3-year for LIHC patients in TCGA cohort. The predicting model nomogram was constructed by involving the clinical and pathological elements, such as gender, age, histologic grade, pathologic stage, neoplasm staging (T, M, and N stage), and TRIM28 level in Figure 3(a). Based on the clinicopathological characteristics, each patient was assigned a nomogram-based score to predict 1-, 2-, and 3-year survival probability. We found that the nomogram model had perfect performance for predicting the 1-year OS, 2-year OS, and 3-year OS of LIHC patients by calibration curves (Figure 3(b)).

### 3.3. Relationship between TRIM28 Expression and Clinicopathology

**Figure 4:**
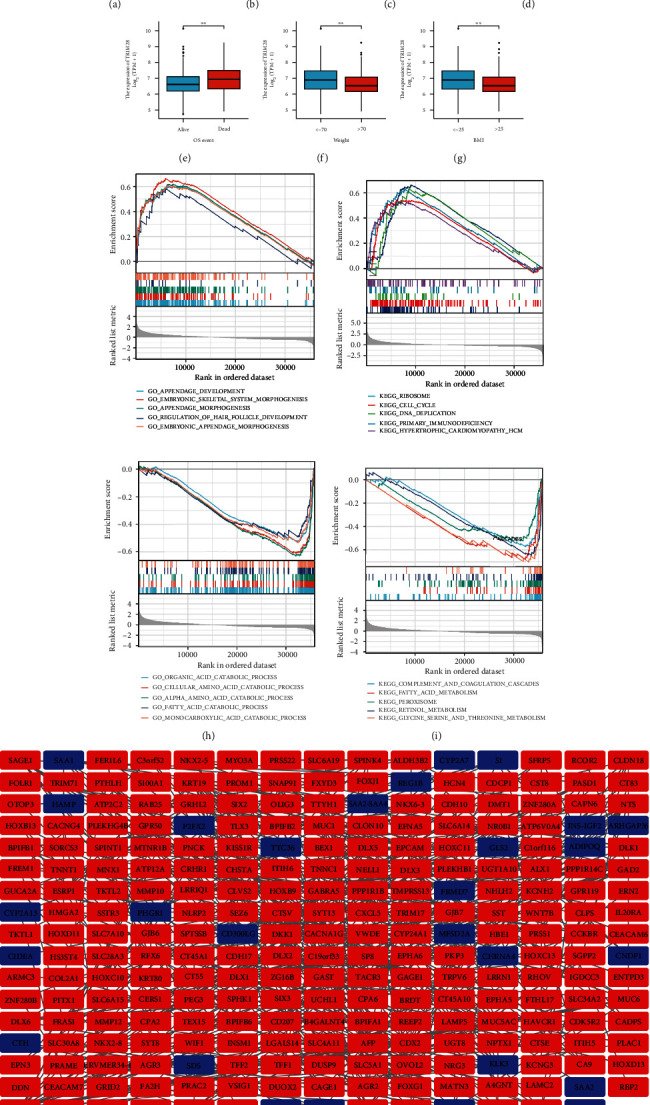
Based on the TCGA data, TRIM28 expression was linked to clinicopathological features of LIHC followed by GO term/KEGG pathway enrichment results. (a) Expression of TRIM28 correlated significantly with T stage. Pathologic stage (b), histologic grade (c), AFP (d), OS event (e), weight (f), and BMI (g). GO term analysis/KEGG pathway revealed five positively correlated groups and five negatively correlated groups (h). (i, j) PPI network. (^∗∗^*P* < 0.01, ^∗∗∗^*P* < 0.001).

As part of the TCGA database, 424 tumor tissues have been analyzed, which includes gene expression data and clinical characteristics collected from patients. LIHC with increased TRIM28 expression was significantly related to the T stage (Figure 4(a)), pathologic stage (Figure 4(b)), histologic grade (Figure 4(c)), AFP (Figure 4(d)), OS event (Figure 4(e)), weight (Figure 4(f)), and BMI (Figure 4(g)). As a result of the study, it was found that high TRIM28 patients had worse T stage, pathologic stage, histological grade, AFP, OS, weight, and worse nutritional status outcomes compared to those with low TRIM28 levels.

### 3.4. GO and KEGG Enrichment Analyses

To better understand how TRIM28 impacts LIHC progression, we then preformed GO term and KEGG analyses by using GSEA. The NES, FDR *Q* value, and nominal *P* value were used to select significantly enriched KEGG pathways and GO terms. As shown in Table 2 and illustrated in Figure 4(h), GO functional analysis showed that these DEGs were associated with positive correlation terms containing appendage development, embryonic skeletal system morphogenesis, appendage morphogenesis, regulation of hair follicle development, and embryonic appendage morphogenesis. According to Table 2 and Figure 4(h), GO functional analysis found that these DEGs play a role in negative correlation terms comprising organic acid catabolic process, cellular amino acid catabolic process, alpha amino acid catabolic process, fatty acid catabolic process, and monocarboxylic acid catabolic process.

As demonstrated in Table 2 and illustrated in Figure 4(i), KEGG enrichment analysis uncovered that these DEGs were concerned with positive correlation terms containing ribosome, cell cycle, DNA replication, primary immunodeficiency, and hypertrophic cardiomyopathy (HCM). As demonstrated in Table 2 and depicted in Figure 4(i), KEGG enrichment analysis revealed these DEGs to be involved in negative correlation terms comprising complement and coagulation cascades, fatty acid metabolism, peroxisome, retinol metabolism, glycine serine, and threonine metabolism. These results suggest that cell cycle, amino acid, and fatty acid metabolism which are critical in LIHC patients, were closely associated with TRIM28 expression.

### 3.5. PPI Network Construction

A total of 606 DEGs were included in the PPI network via the STRING database. Using 254 nodes and 471 edges, the PPI network was constructed to examine the interactions of DEGs correlated with LIHC risk.

In STRING database, 606 DEGs were incorporated into the PPI network in total, including 254 nodes and 471 edges, which could examine the interaction of DEGs related to LIHC risk. (Figure 4(j)).

### 3.6. Relationship between TRIM28 Expression and Tumor-Infiltrating Immune Cells

**Figure 5:**
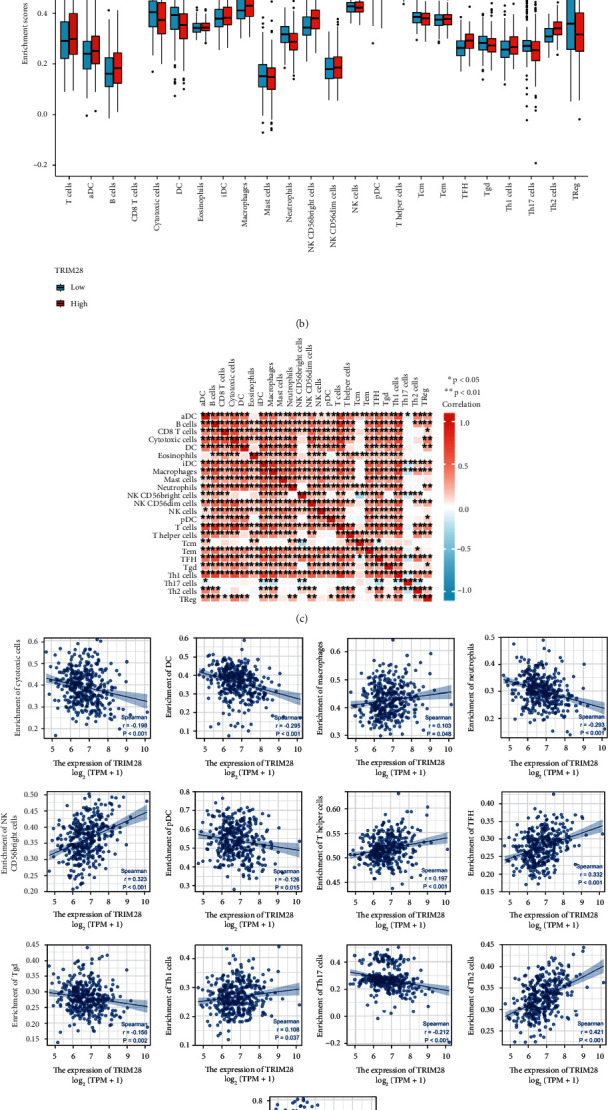
The analysis of TRIM28 expression in LIHC correlated with immune infiltration levels by TIMER. (a) The relationship between TRIM28 expression and immune infiltration. (b) The varied proportions of 24 subtypes of immune cells in high and low TRIM28 expression groups in tumor samples. (c) The heat map shows the infiltration of 24 immune cells into tumor samples. (d) The correlation between TRIM28 and immunocytes was calculated via ssGSEA.

Tumor-infiltrating lymphocytes (TILs) were considered to be an independent predictor of OS and sentinel lymph node status in cancer. Therefore, we analyzed the relationship between TRIM28 and immune infiltration level by selecting the TIMER (Figure 5(a)). It was found that there was a positive correlation between TRIM28 expression levels and B cell (*P* value = 1.98 × 10^−17^), CD8+ T cell (*P* value = 2.15 × 10^−6^), CD4+ T cell (*P* value = 4.79 × 10^−10^), macrophage (*P* value = 6.45 × 10^−13^), neutrophil (*P* value = 1.39 × 10^−6^), and dendritic cell (*P* value = 1.83 × 10^−12^). Based on above results, an important and pivotal role was played by TRIM28 in immune infiltration. Our study also sought to determine if there was a difference in tumor immune microenvironment between LIHC patients with low TRIM28 levels and patients with high TRIM28 levels. Analyzing the difference between 24 immune cells with high and low TRIM28 expression was done using the CIBERSORT algorithm (Figure 5(b)). A significant difference between high and low TRIM28 levels existed in T cell lines, with B cells, cytotoxic cells, dendritic cell (DC), neutrophils, CD56bright NK cells, T helper cells, T follicular helper cells (TFH), T *γδ* (gamma delta) cells (Tgd), helper T type 1 (Th1) cells, helper T type 2 (Th2) cells, helper T type 17 (Th17) cells, and T regulatory cell (Treg) influenced by TRIM28 levels. B cells, NK CD56bright cells, T helper cells, TFH, Th1 cells, Th2 cells were increased compared to the group with low TRIM28 expression while cytotoxic cells, DC, neutrophils, Tgd, Th17 cells, and Treg were decreased the group with high TRIM28 expression. The further investigation of possible correlations between 24 immune cell types was performed (Figure 5(c)). According to the heat map, there were moderate to strong correlations between subpopulations of TIICs. Finally, the relevance between TRIM28 and other immunocytes was assessed for yet again by using the GSVA package (Figure 5(d)).

### 3.7. Data Validation

**Figure 6:**
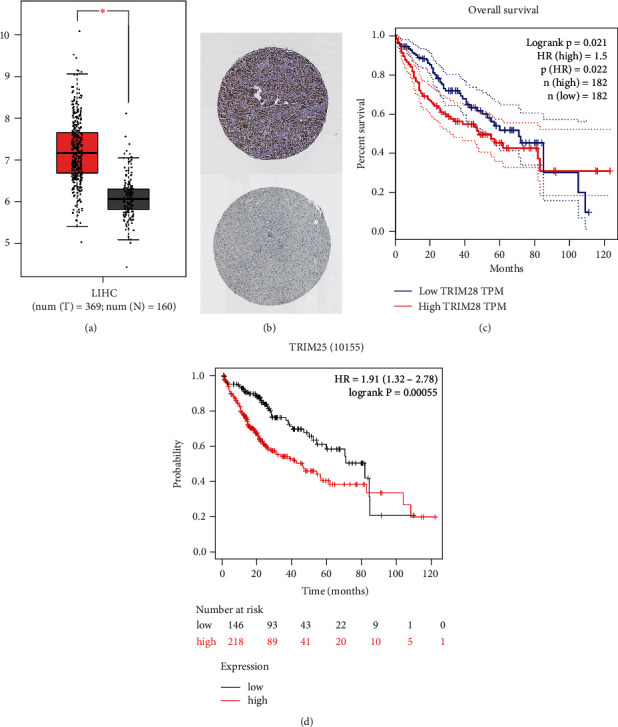
Synthesized analysis of TRIM28 mRNA expression and prognosis in patients with LIHC. (a) From GEPIA, TRIM28 mRNA expression levels in normal and LIHC tissues. (b) Hepatic expression of TRIM28 protein was visualized using immunohistochemistry via THPA. (c) The level of expression of TRIM28 mRNA and OS as determined by GEPIA. (d) Validation of the correlation between TRIM28 expression and OS based on K-M survival plots.

First, we used the GEPIA database to analyze TRIM28 expression. The TRIM28 expression was increased in the LIHC group in Figure 6(a). The immunohistochemistry (IHC) images also showed that TRIM28 was more expressed in tumor tissues than in nontumor tissues (Figure 6(b)). There was a significant correlation between high TRIM28 level and poor OS for LIHC (*P* value = 0.021 < 0.05, Figure 6(c)). In addition, we performed K-M survival plots to confirm this result. As shown in Figure 6(d), the K-M survival plots revealed that high TRIM28 expression groups had a significant correlation with poor OS rates (*P* value = 0.00055 < 0.05).

### 3.8. TRIM28 Possesses a Higher Specificity than AFP for LIHC Diagnosis and qRT-PCR for an External Validation

**Figure 7:**
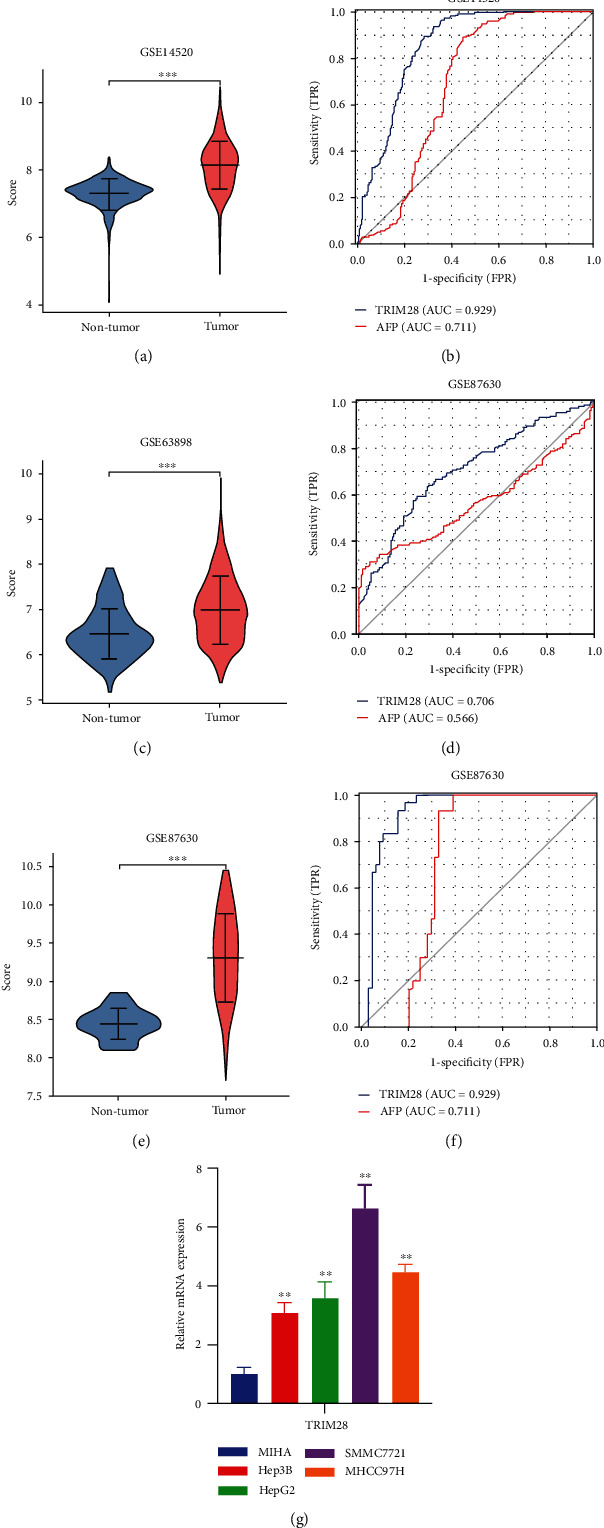
GEO and qRT-PCR verification of the diagnostic value of TRIM28 in LIHC patients. (a) Violin plot showing TRIM28 mRNA levels in patients with nontumor (*n* = 220) and LIHC (*n* = 225) on the GSE14520 dataset. (b) ROC curve for LIHC on the GSE14520 dataset. (c) Violin plot showing TRIM28 mRNA levels in patients with nontumor (*n* = 168) and LIHC (*n* = 228) from the GSE63898 dataset. (d) ROC curve for LIHC on the GSE63898 dataset. (e) Violin plot showing TRIM28 mRNA levels in patients with nontumor (*n* = 30) and LIHC (*n* = 64) from the GSE87630 dataset. (f) ROC curve for LIHC on the GSE87630 dataset. (g) TRIM28 expression level in LIHC by using qRT-PCR.

As a final step in evaluating the diagnostic value of TRIM28, we used ROC analysis to analyze GSE14520, GSE63898, and GSE87630 datasets. LIHC is commonly associated with AFP, a diagnostic tumor marker. The TRIM28 expression in GSE14520 was significantly higher than nontumor tissue (Figure 7(a)), and the AUC of TRIM28 in GSE14520 was 0.853, which was higher than that of AFP (0.685) (Figure 7(b)). In GSE63898, the expression of TRIM28 in nontumor tissues was notably lower than that in tumor tissues (Figure 7(c)). The AUC value of AFP in this dataset was 0.566, which was lower than that of TRIM28(0.706) (Figure 7(d)). The TRIM28 expression in GSE87630 was obviously higher than nontumor tissue (Figure 7(e)), and the AUC of AFP was 0.711, which was lower than that of TRIM28 (0.929) (Figure 7(f)). In Figure 7(g), qRT-PCR was performed on multiple cell lines to confirm TRIM28 expression. These results demonstrated that TRIM28 could be useful as a diagnostic marker for LIHC patients.

## 4. Discussion

Cancer deaths from LIHC are the 3rd leading cause of death from cancer, and it is one of the five most commonly diagnosed types [33]. The prevalence of LIHC has continued to increase over the past two decades [34]. Prevention and treatment are essential for the survival of this devastating disease. Bioinformatics analysis was found to be the most suitable solution [35]. A number of previous biomarker studies have proved the effectiveness of this method in LIHC. It was recently discovered that MAST2 is a biomarker that can be used to diagnose and prognosis LIHC by dry-lab analyses. There was a correlation between high MAST2 and late clinical state [36].

As part of our research on LIHC, we were evaluating TRIM28 as a prognostic biomarker. By analyzing the TCGA database, we evaluated TRIM28's prognostic value for patients with LIHC. Further analysis showed that TRIM28 was an independent prognostic factor, and the higher the expression of TRIM28, the worse the survival rate. A high expression level of TRIM28 was associated with the following factors: T classification, pathologic stage, histologic grade, AFP, OS event, weight, and BMI. In conclusion, these results suggest that the expression level of TRIM28 may affect the occurrence, development, and immune microenvironment of LIHC.

GO and KEGG pathway analyses revealed that TRIM28 was participated in cell cycle, amino acid, and fatty acid metabolism. The function of cell cycle pathways in the regulation of tumor had been previously demonstrated [37].

Immunomodulation by antitumor cell cycle inhibitors could be the promising targets of cancer therapy [38–40]. At the same time, amino acid and fatty acid metabolism also were considered as a potential targeted therapeutic strategy for cancer therapy [41, 42]. Studies have shown that fatty acid receptor and synthase represent a potential strategy and attractive target for tumor treatment [43]. Furthermore, all of those pathways are both recognized as playing a major role in tumor immunity [44–47]. As a next step, we investigated the relationship between TRIM28 and immune cell infiltration.

The purpose of this study was to examine the relationship between TRIM28 and immuno-cell infiltration level in LIHC by using the TIMER database. It was found that TRIM28 was positively related with B cell, CD8+ T Cell, CD4+ T Cell, macrophage, neutrophil, and dendritic cell. By the CIBERSORT algorithm, we confirmed that high TRIM28 expression was related with upregulation of CD56bright NK cells, T helper cells, TFH, Th1 cells, Th2 cells and downregulation of cytotoxic cells, DC, neutrophils, Tgd, Th17 cells, and Treg. Among the functionally specialized antigen-presenting cells, DC played an important role in innate antitumor immunity by activating specific T cells [48]. Tumor development was also inhibited by DC through regulation of humoral immune responses [49]. Secondly, cytotoxic cells and neutrophils played an important role in killing tumor cells [50, 51]. As such, our hypothesis was that TRIM28 overexpression could reduce the activity of DCs, cytotoxic cells, and neutrophils. As a result of these studies, TRIM28 is critical for modulating LIHC immune responses. The mechanism by which TRIM28 promotes LIHC immune responses activation is unclear; however, it is necessary to conduct multicenter, randomized, controlled clinical trials, and mechanism studies to better understand the relationship between TRIM28 and LIHC [52–56].

As a final step, GEO datasets and its ROC curve analysis are used to validate our results. Meanwhile, qRT-PCR was used to further verify the expression of TRIM28 in multiple cell lines. It was found that expression levels of TRIM28 were higher than those of nontumor tissues and AUC values of TRIM28 also were higher than those of AFP, the mainstream biomarker for LIHC in 3 datasets. It was demonstrated that liver cancer cell lines expressing TRIM28 were highly expressed. In summary, these results showed TRIM28 was a positive predictive tumor marker for LIHC patients.

Several drawbacks remain in our study. With regard to the first point, let us look at data sources that are sourced from public databases. We solely validated the result by using qRT-PCR in cell lines. Sufficient serum samples from clinical patients will be necessary for the validation of these biomarkers in the future. Next, we will discuss the 2nd point. Because the effectiveness of markers is considerably dependent on mechanism, we need to validate the hypothesis experimentally and elucidate its mechanisms in cancer cells by siRNA or plasmid. Moreover, transgenic animal research is needed to further verify the TRIM28 functions. Fortunately, we have demonstrated that TRIM28 was highly expressed in liver cancer cells line. There was sufficient evidence to initiate further study.

## 5. Conclusions

Based on bioinformatic analysis and qRT-PCR, TRIM28 associated with LIHC has been identified. There is a novel and independent prognostic LIHC biomarker, TRIM28, that correlates with immune infiltrates. The TRIM28 gene will provide a novel perspective on LIHC mechanisms with further study in the future. As an effective diagnostic and intervention gene, TRIM28 will be able to diagnose and treat LIHC at an early stage.

## Figures and Tables

**Table 1:** Correlation between overall survival and multivariable characteristics in TCGA patients via Cox regression and multivariate survival model.

Characteristics	Total (*n*)	Univariate analysis		Multivariate analysis	
		Hazard ratio (95% CI)	*P* value	Hazard ratio (95% CI)	*P* value
Age	373				
≤60	177	Reference			
>60	196	1.205 (0.850-1.708)	0.295	1.396 (0.855-2.281)	0.183
Gender	373				
Female	121	Reference			
Male	252	0.793 (0.557-1.130)	0.200	0.945 (0.574-1.555)	0.823
Histologic grade	368				
G1 & G2	233	Reference			
G3 & G4	135	1.091 (0.761-1.564)	0.636	0.968 (0.589-1.589)	0.897
Pathologic stage	349				
Stage I & stage II	259	Reference			
Stage III & Stage IV	90	2.504 (1.727-3.631)	**<0.001**	0.421 (0.023-7.620)	0.559
T stage	370				
T1 & T2	277	Reference			
T3 & T4	93	2.598 (1.826-3.697)	**<0.001**	6.493 (0.370-113.852)	0.200
M stage	272				
M0	268	Reference			
M1	4	4.077 (1.281-12.973)	**0.017**	1.790 (0.513-6.244)	0.361
N stage	258				
N0	254	Reference			
N1	4	2.029 (0.497-8.281)	0.324	3.099 (0.406-23.637)	0.275
TRIM28	373	1.578 (1.272-1.958)	**<0.001**	1.687 (1.252-2.275)	**<0.001**

**(a):** 

	GO NAME	NES	NOM *p*-value	FDR *p*-value
Positive	GO_APPENDAGE_DEVELOPMENT	2.415	0.001	0.031
	GO_EMBRYONIC_SKELETAL_SYSTEM_MORPHOGENESIS	2.398	0.001	0.031
	GO_APPENDAGE_MORPHOGENESIS	2.379	0.001	0.031
	GO_REGULATION_OF_HAIR_FOLLICLE_DEVELOPMENT	2.314	0.002	0.032
	GO_EMBRYONIC_APPENDAGE_MORPHOGENESIS	2.301	0.001	0.031

Negative	GO_ORGANIC_ACID_CATABOLIC_PROCESS	-3.277	0.016	0.079
	GO_CELLULAR_AMINO_ACID_CATABOLIC_PROCESS	-3.095	0.007	0.052
	GO_ALPHA_AMINO_ACID_CATABOLIC_PROCESS	-3.062	0.006	0.046
	GO_FATTY_ACID_CATABOLIC_PROCESS	-3.045	0.006	0.048
	GO_MONOCARBOXYLIC_ACID_CATABOLIC_PROCESS	-3.001	0.008	0.054

**(b):** 

	KEGG NAME	NES	NOM *p*-value	FDR *p*-value
Positive	KEGG_RIBOSOME	2.301	0.001	0.036
	KEGG_CELL_CYCLE	1.969	0.001	0.036
	KEGG_DNA_REPLICATION	1.885	0.001	0.036
	KEGG_PRIMARY_IMMUNODEFICIENCY	1.865	0.001	0.036
	KEGG_HYPERTROPHIC_CARDIOMYOPATHY_HCM	1.822	0.002	0.036

Negative	KEGG_COMPLEMENT_AND_COAGULATION_CASCADES	-2.972	0.004	0.038
	KEGG_FATTY_ACID_METABOLISM	-2.802	0.004	0.036
	KEGG_PEROXISOME	-2.795	0.005	0.040
	KEGG_RETINOL_METABOLISM	-2.683	0.005	0.038
	KEGG_GLYCINE_SERINE_AND_THREONINE_METABOLISM	-2.571	0.003	0.036

## Data Availability

The gene expression profiling data supporting this study are from previously reported studies and datasets, which have been cited. The expression and survival data are derived from TCGA and GEO databases. TCGA and GEO belong to public databases. The patients involved in the database have obtained ethical approval. Users can download relevant data for free for research and publish relevant articles. The qRT-PCR data used to support the findings of this study have not been made available. (https://www.ncbi.nlm.nih.gov/geo/query/acc.cgi?acc=GSE14520; https://www.ncbi.nlm.nih.gov/geo/query/acc.cgi?acc=GSE63898; and https://www.ncbi.nlm.nih.gov/geo/query/acc.cgi?acc=GSE87630).

## Supplementary Materials

Supplementary Materials The flow chart of the present experiment was performed in supplementary figure 1. Supplementary figure 1: The overall implementation framework of the article.

## References

[1] Maluccio M., Covey A. (2012). Recent progress in understanding, diagnosing, and treating hepatocellular carcinoma. *CA: a Cancer Journal for Clinicians*.

[2] Wang Y., Luo G., Shen M. (2020). The effect of meditative movement on the quality of life in patients recovering from COVID-19: a protocol for systematic review and meta-analysis. *Medicine*.

[3] Wang Y., Han J., Zhou H., Ai S., Wan D. (2022). A prognosis marker dynein cytoplasmic 1 heavy chain 1 correlates with EMT and immune signature in liver hepatocellular carcinoma by bioinformatics and experimental analysis. *Disease Markers*.

[4] Qiang R., Zhao Z., Tang L., Wang Q., Wang Y., Huang Q. (2021). Identification of 5 hub genes related to the early diagnosis, tumour stage, and poor outcomes of hepatitis B virus-related hepatocellular carcinoma by bioinformatics analysis. *Computational and Mathematical Methods in Medicine*.

[5] Lee Y., Moon H., Lee J. (2021). Association of metabolic risk factors with risks of cancer and all-cause mortality in patients with chronic hepatitis B. *Hepatology (Baltimore, Md)*.

[6] McMahon B., Nolen S., Snowball M. (2021). HBV genotype: a significant risk factor in determining which patients with chronic HBV infection should undergo surveillance for HCC: the hepatitis B Alaska study. *Hepatology (Baltimore, Md)*.

[7] Huang D., El-Serag H., Loomba R. (2021). Global epidemiology of NAFLD-related HCC: trends, predictions, risk factors and prevention. *Nature Reviews Gastroenterology & Hepatology*.

[8] Gan W., Huang J., Zhang M. (2018). New nomogram predicts the recurrence of hepatocellular carcinoma in patients with negative preoperative serum AFP subjected to curative resection. *Journal of Surgical Oncology*.

[9] Jefferies C., Wynne C., Higgs R. (2011). Antiviral TRIMs: friend or foe in autoimmune and autoinflammatory disease?. *Nature Reviews Immunology*.

[10] Chikuma S., Suita N., Okazaki I., Shibayama S., Honjo T. (2012). TRIM28 prevents autoinflammatory T cell development *in vivo*. *Nature Immunology*.

[11] Jin J., Lee G., Nam S. (2021). Sequential ubiquitination of p53 by TRIM28, RLIM, and MDM2 in lung tumorigenesis. *Cell Death and Differentiation*.

[12] Lionnard L., Duc P., Brennan M. (2019). TRIM17 and TRIM28 antagonistically regulate the ubiquitination and anti- apoptotic activity of BCL2A1. *Cell Death and Differentiation*.

[13] Park H., Kim H., Park S. (2021). RIPK3 activation induces TRIM28 derepression in cancer cells and enhances the anti-tumor microenvironment. *Molecular Cancer*.

[14] Wang Y., Wang Q., Li X. (2021). Paeoniflorin sensitizes breast cancer cells to tamoxifen by downregulating microRNA-15b via the FOXO1/CCND1/*β*-catenin axis. *Drug Design, Development and Therapy*.

[15] Liu J., Han X., Chen L. (2020). *TRIM28* is a distinct prognostic biomarker that worsens the tumor immune microenvironment in lung adenocarcinoma. *Aging*.

[16] Czerwinska P., Jaworska A., Wlodarczyk N., Mackiewicz A. A. (2020). Melanoma stem cell-like phenotype and significant suppression of immune response within a tumor are regulated by TRIM28 protein. *Cancers*.

[17] Czerwinska P., Mackiewicz A. (2021). Low levels of TRIM28-interacting KRAB-ZNF genes associate with cancer stemness and predict poor prognosis of kidney renal clear cell carcinoma patients. *Cancers*.

[18] Fitzgerald S., Espina V., Liotta L. (2018). Stromal TRIM28-associated signaling pathway modulation within the colorectal cancer microenvironment. *Journal of Translational Medicine*.

[19] Wang Z., Jensen M., Zenklusen J. (2016). A practical guide to the cancer genome atlas (TCGA). *Methods in Molecular Biology (Clifton, NJ)*.

[20] Haussler D. (2013). The cancer genome atlas. *Science*.

[21] Roessler S., Jia H., Budhu A. (2010). A unique metastasis gene signature enables prediction of tumor relapse in early-stage hepatocellular carcinoma patients. *Cancer Research*.

[22] Villanueva A., Portela A., Sayols S. (2015). DNA methylation-based prognosis and epidrivers in hepatocellular carcinoma. *Hepatology (Baltimore, Md)*.

[23] Woo H., Choi J., Yoon S. (2017). Integrative analysis of genomic and epigenomic regulation of the transcriptome in liver cancer. *Nature Communications*.

[24] Robin X., Turck N., Hainard A. (2011). pROC: an open-source package for R and S+ to analyze and compare ROC curves. *BMC Bioinformatics*.

[25] Subramanian A., Tamayo P., Mootha V. (2005). Gene set enrichment analysis: a knowledge-based approach for interpreting genome-wide expression profiles. *Proceedings of the National Academy of Sciences of the United States of America*.

[26] Szklarczyk D., Gable A., Nastou K. (2021). The STRING database in 2021: customizable protein-protein networks, and functional characterization of user-uploaded gene/measurement sets. *Nucleic Acids Research*.

[27] Shannon P., Markiel A., Ozier O. (2003). Cytoscape: a software environment for integrated models of biomolecular interaction networks. *Genome Research*.

[28] Li T., Fan J., Wang B. (2017). TIMER: a web server for comprehensive analysis of tumor-infiltrating immune cells. *Cancer Research*.

[29] Aran D., Sirota M., Butte A. (2015). Systematic pan-cancer analysis of tumour purity. *Nature Communications*.

[30] Newman A. M., Liu C. L., Green M. R. (2015). Robust enumeration of cell subsets from tissue expression profiles. *Nature Methods*.

[31] Tang Z., Li C., Kang B., Gao G., Li C., Zhang Z. (2017). GEPIA: a web server for cancer and normal gene expression profiling and interactive analyses. *Nucleic Acids Research*.

[32] Uhlén M., Fagerberg L., Hallström B. (2015). Proteomics. Tissue-based map of the human proteome. *Science (New York, N.Y.)*.

[33] An L., Zeng H. M., Zheng R. S. (2019). Liver cancer epidemiology in China, 2015. *Zhonghua Zhong liu za zhi [Chinese Journal of Oncology]*.

[34] Frenette C. (2020). Advances in hepatocellular carcinoma. *Clinics in Liver Disease*.

[35] Han J., Wang Y., Zhou H., Zhang Y., Wan D. (2022). CD137 regulates bone loss via the p53 Wnt/*β*-catenin signaling pathways in aged mice. *Frontiers in Endocrinology*.

[36] Jiao Y., Li Y., Jiang P., Fu Z., Liu Y. (2019). High MAST2 mRNA expression and its role in diagnosis and prognosis of liver cancer. *Scientific Reports*.

[37] Matthews H., Bertoli C., de Bruin R. (2022). Cell cycle control in cancer. *Nature Reviews Molecular Cell Biology*.

[38] Ingham M., Schwartz G. (2017). Cell-cycle therapeutics come of age. *Journal of Clinical Oncology : Official Journal of the American Society of Clinical Oncology*.

[39] Otto T., Sicinski P. (2017). Cell cycle proteins as promising targets in cancer therapy. *Nature Reviews Cancer*.

[40] Petroni G., Formenti S., Chen-Kiang S., Galluzzi L. (2020). Immunomodulation by anticancer cell cycle inhibitors. *Nature Reviews Immunology*.

[41] Dierge E., Larondelle Y., Feron O. (2020). Cancer diets for cancer patients: lessons from mouse studies and new insights from the study of fatty acid metabolism in tumors. *Biochimie*.

[42] Li Z., Zhang H. (2016). Reprogramming of glucose, fatty acid and amino acid metabolism for cancer progression. *Cellular and Molecular Life Sciences : CMLS*.

[43] Fhu C., Ali A. (2020). Fatty acid synthase: an emerging target in cancer. *Molecules (Basel, Switzerland)*.

[44] Wang W., Zou W. (2020). Amino acids and their transporters in T cell immunity and cancer therapy. *Molecular Cell*.

[45] Yoon H., Lee S. (2022). Fatty acid metabolism in cvarian cancer: therapeutic implications. *International Journal of Molecular Sciences*.

[46] Siddiqui S., Glauben R. (2022). Fatty acid metabolism in myeloid-derived suppressor cells and tumor-associated macrophages: key factor in cancer immune evasion. *Cancers (Basel)*.

[47] Wang Y., Wu B., Ai S., Wan D. (2022). Electroplating of HAp-brushite coating on metallic bioimplants with advanced hemocompatibility and osteocompatibility properties. *Journal of Applied Biomaterials & Functional Materials*.

[48] Zhu S., Yang N., Wu J. (2020). Tumor microenvironment-related dendritic cell deficiency: a target to enhance tumor immunotherapy. *Pharmacological Research*.

[49] Lurje I., Hammerich L., Tacke F. (2020). Dendritic cell and T cell crosstalk in liver fibrogenesis and hepatocarcinogenesis: implications for prevention and therapy of liver cancer. *International Journal of Molecular Sciences*.

[50] Raskov H., Orhan A., Christensen J., Gögenur I. (2021). Cytotoxic CD8^+^ T cells in cancer and cancer immunotherapy. *British Journal of Cancer*.

[51] Tolle F., Umansky V., Utikal J., Kreis S., Bréchard S. (2021). Neutrophils in tumorigenesis: missing targets for successful next generation cancer therapies?. *International Journal of Molecular Sciences*.

[52] Su Y., Wan D., Song W. (2016). Dryofragin inhibits the migration and invasion of human osteosarcoma U2OS cells by suppressing MMP-2/9 and elevating TIMP-1/2 through PI3K/AKT and p38 MAPK signaling pathways. *Anti-Cancer Drugs*.

[53] Wan D., Jiang C., Hua X., Wang T., Chai Y. (2015). Cell cycle arrest and apoptosis induced by aspidin PB through the p53/p21 and mitochondria-dependent pathways in human osteosarcoma cells. *Anti-Cancer Drugs*.

[54] Wang Y., Wan D., Zhou R., Zhong W., Lu S., Chai Y. (2017). Geraniin inhibits migration and invasion of human osteosarcoma cancer cells through regulation of PI3K/Akt and ERK1/2 signaling pathways. *Anti-Cancer Drugs*.

[55] Wan D., Qu Y., Zhang L., Ai S., Cheng L. (2020). The lncRNA LINC00691 functions as a ceRNA for miRNA-1256 to suppress osteosarcoma by regulating the expression of ST5. *Oncotargets and Therapy*.

[56] Yu T., Chen D., Zhang L., Wan D. (2019). microRNA-26a-5p promotes proliferation and migration of osteosarcoma cells by targeting *HOXA5* in vitro and in vivo. *Oncotargets and Therapy*.

