# Supplementary figures and images for: Integrated Bioinformatics and Experimental Analysis Identified TRIM28 a Potential Prognostic Biomarker and Correlated with Immune Infiltrates in Liver Hepatocellular Carcinoma
Source: Comput Math Methods Med. 2022 Oct 4;2022:6267851. doi: 10.1155/2022/6267851 (PMC9553339; doi:10.1155/2022/6267851)

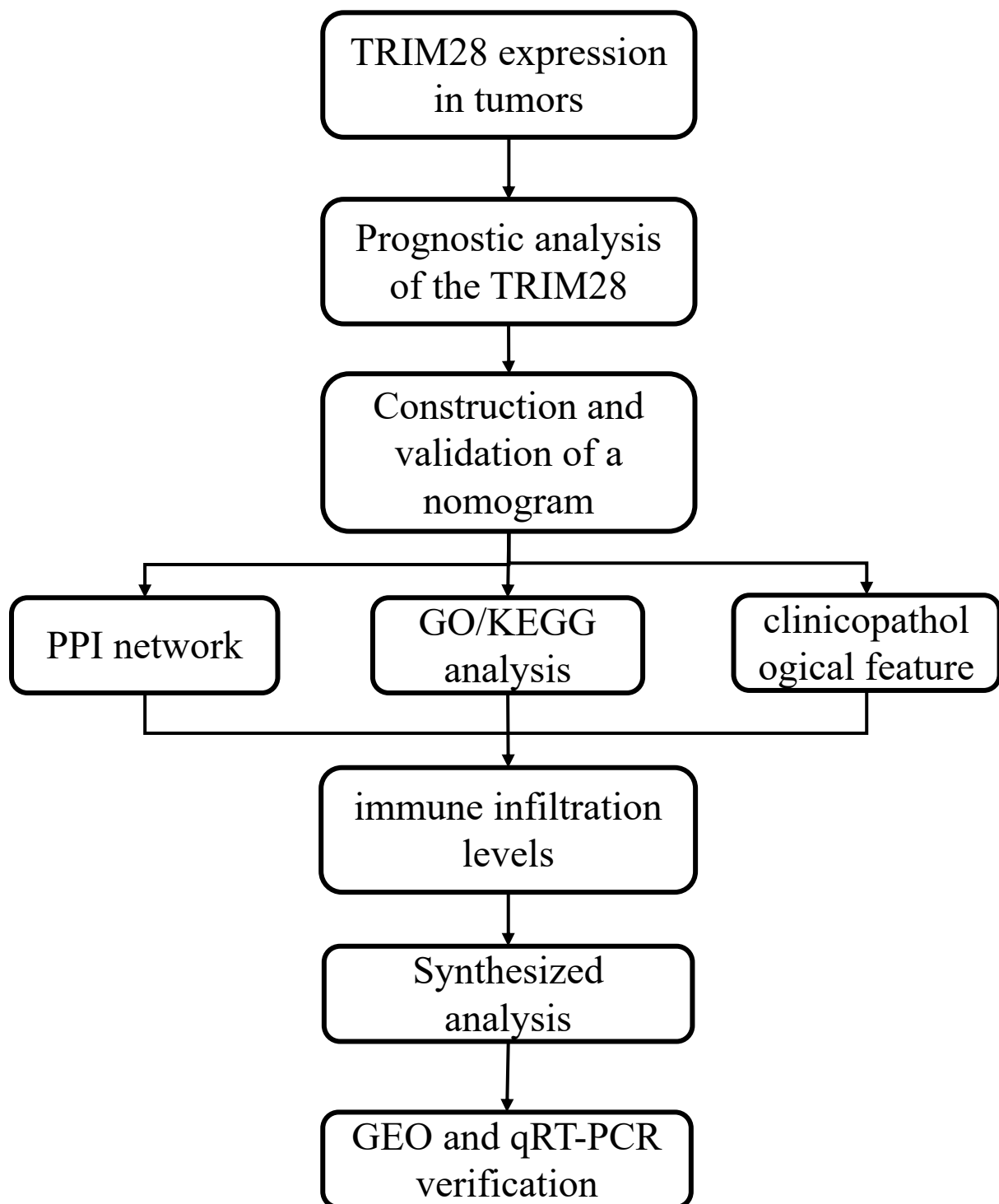

Supplement: Supplementary Materials — The flow chart of the present experiment was performed in supplementary figure 1. Supplementary figure 1: The overall implementation framework of the article. [file 6267851.f1.pdf]
